# Successes, weaknesses, and recommendations to strengthen primary health care: a scoping review

**DOI:** 10.1186/s13690-023-01116-0

**Published:** 2023-06-02

**Authors:** Aklilu Endalamaw, Daniel Erku, Resham B. Khatri, Frehiwot Nigatu, Eskinder Wolka, Anteneh Zewdie, Yibeltal Assefa

**Affiliations:** 1 School of Public Health, The University of Queensland, Brisbane, Australia; 2 College of Medicine and Health Sciences, Bahir Dar University, Bahir Dar, Ethiopia; 3 Centre for Applied Health Economics, School of Medicine, Griffith University, Brisbane, Australia; 4 Menzies Health Institute Queensland, Griffith University, Brisbane, Australia; 5 Health Social Science and Development Research Institute, Kathmandu, Nepal; 6 International Institute for Primary Health Care in Ethiopia, Addis Ababa, Ethiopia

**Keywords:** Primary health care, Primary care, Successes, Weaknesses, Strategies, Barriers, Scoping Review

## Abstract

**Background:**

Primary health care (PHC) is a roadmap for achieving universal health coverage (UHC). There were several fragmented and inconclusive pieces of evidence needed to be synthesized. Hence, we synthesized evidence to fully understand the successes, weaknesses, effective strategies, and barriers of PHC.

**Methods:**

We followed the PRISMA extension for scoping reviews checklist. Qualitative, quantitative, or mixed-approach studies were included. The result synthesis is in a realistic approach with identifying which strategies and challenges existed at which country, in what context and why it happens.

**Results:**

A total of 10,556 articles were found. Of these, 134 articles were included for the final synthesis. Most studies (86 articles) were quantitative followed by qualitative (26 articles), and others (16 review and 6 mixed methods). Countries sought varying degrees of success and weakness. Strengths of PHC include less costly community health workers services, increased health care coverage and improved health outcomes. Declined continuity of care, less comprehensive in specialized care settings and ineffective reform were weaknesses in some countries. There were effective strategies: leadership, financial system, ‘Diagonal investment’, adequate health workforce, expanding PHC institutions, after-hour services, telephone appointment, contracting with non-governmental partners, a ‘Scheduling Model’, a strong referral system and measurement tools. On the other hand, high health care cost, client’s bad perception of health care, inadequate health workers, language problem and lack of quality of circle were barriers.

**Conclusions:**

There was heterogeneous progress towards PHC vision. A country with a higher UHC effective service coverage index does not reflect its effectiveness in all aspects of PHC. Continuing monitoring and evaluation of PHC system, subsidies to the poor, and training and recruiting an adequate health workforce will keep PHC progress on track. The results of this review can be used as a guide for future research in selecting exploratory and outcome parameters.

**Supplementary Information:**

The online version contains supplementary material available at 10.1186/s13690-023-01116-0.

## Background

A comprehensive primary health care (PHC) allows all members of the population to access essential health services without financial catastrophe [1] that is given in district hospitals, health centres, clinics and health posts [2–4]. PHC is a ‘whole system approach’—to deliver health promotion, disease prevention, curative and rehabilitative care—supported by medical supplies, multidisciplinary health teams, health governance and financing [5–7]. Moreover, it delivers health care services which have gotten attention since 1978 at ‘Alma-Ata’ declaration [8] and other prioritized services through time, like public health emergencies, common eye-nose-throat and oral health problems and mental health services [7, 9, 10].

PHC in its first inception aimed for ‘Health for All by the Year 2000’. Eventually, PHC is amenable to any global and national health policies, and most recently, it is a roadmap for achieving universal health coverage (UHC) by 2030 [11]. As a result, the global leaders and country representatives proclaimed a renewed action on PHC towards UHC in an international conference held in Kazakhstan, in October 2018 [12].

However, the World Health Organisation (WHO) projected that only 39% to 63% of the global population would be covered for essential health services by 2030 [13]. Hence, to take corrective actions and support government investment in PHC, health policy needs evidence about the challenges and effective strategies. In 2013, a review paper reported the impact of PHC delivery models [14] that discussed PHC models in improving access, quality and care coordination. However, it did not address PHC success, strategies, weaknesses, or challenges. Capacity building, human resources for health, technology, financing, and empowering individuals and communities complement the health system [8, 12, 15–18].

This study synthesized successes, strategies, weakness, and barriers of PHC dimensions. Therefore, the current study’s findings will be crucial to supplement PHC-related policy design, implementation, and evaluation.

## Methods

### Reporting

The review was conducted per Levac and colleagues’ [19] five-step approach, including identifying research questions, identifying and selecting relevant studies, extracting data, and summarizing and reporting results. In addition, we followed the PRISMA extension for scoping reviews checklist to report this review (Additional file).

### Search strategy

The required data were collected by searching on 4 May 2022 in the PubMed database and hand search by using the Google Scholar search engine. The search was updated on 28 April 2023. The key search terms or phrases used for searching articles fitted to PubMed were ("primary health care"[Title]) OR ("primary healthcare"[Title]) OR ("primary health-care"[Title]) Filters: English.

### Inclusion and exclusion criteria

We included all types of articles that evaluated primary health care. These articles are quantitative, qualitative, mixed, or review by using data from clients, communities, document or article reviews, or health institutions. The types of articles were identified during the screening and data-extraction phase. Quantitative articles are estimated and presented the results mathematically, while qualitative articles are perspectives, in-depth interviews, focus-group discussions, and observations in which results are presented in texts. A review was any types of one or more principles of PHC. We considered mixed studies when quantitative and qualitative approaches are integrated into a single study. Since primary care is a subset of primary health care, we focused on the core principles of primary care in this synthesis. When the success and weakness of PHC researched its core principles i.e., accessibility, quality of care, effectiveness, cost-effectiveness, coordination, continuity, comprehensiveness, efficiency, equity and patient-centredness, we included all these as well. There were no time and place restrictions.

Articles with abstract or title only, letters to editors, perspectives, commentaries, conference abstracts and studies that do not have reported relevant findings to the current objectives were excluded. Articles published other than in English were also excluded.

### Study selection and data extraction

Title, abstract and full-text screening was conducted by two authors (AE, DE) and the third author was involved whenever disagreement happened (YA). Then, appropriate data was extracted from included articles. These are: first author, publication year, country (study setting), study approach, study population, attributes, and objectives are displayed in the supplementary file (Table S1), and the main findings are presented in the result section.

### Statistical analysis and synthesis

UHC effective service coverage index of countries mentioned in the included articles are presented using the Choropleth map. We generated Choropleth map using R-software. Data of UHC effective service coverage index was taken from the Global health observatory [20]. UHC effective service coverage index is a composite of a single summary indicator estimated from the coverage value of 14 tracer indicators, mainly from infectious diseases (tuberculosis and HIV/AIDS); reproductive, maternal, neonatal and child health services; non-communicable disease treatments (hypertension control); service capacity and access [20]. As a ‘whole-of-society’ context, leadership, financial system, human resources and other facilitators or barriers were identified. The result synthesis is in a realistic approach i.e., showing which strategies and challenges were identified in which country, in what context and why it happens. Then, the strategies and barriers of PHC dimensions is summerised in figure.

## Results

### Search results

Using search strategy, 10,323 articles were found in PubMed (9,466 on 04 May 2022 and 857 on 28 April 2023) and 233 from Google Scholar. A total of 569 remain after title screening. Following excluding title only, abstract only and unrelated abstract, 219 were eligible for full-text review. Letters, editorials, commentaries, perspectives, and full-text articles with unrelated findings were screened further. Finally, 134 articles were included for the result synthesis. Most studies (86 articles) were quantitative followed by qualitative (26 articles), and others (16 reviews and 6 mixed methods) (sT1).

### Primary health care success, weakness, strategies, and barriers

**Fig. 1:**
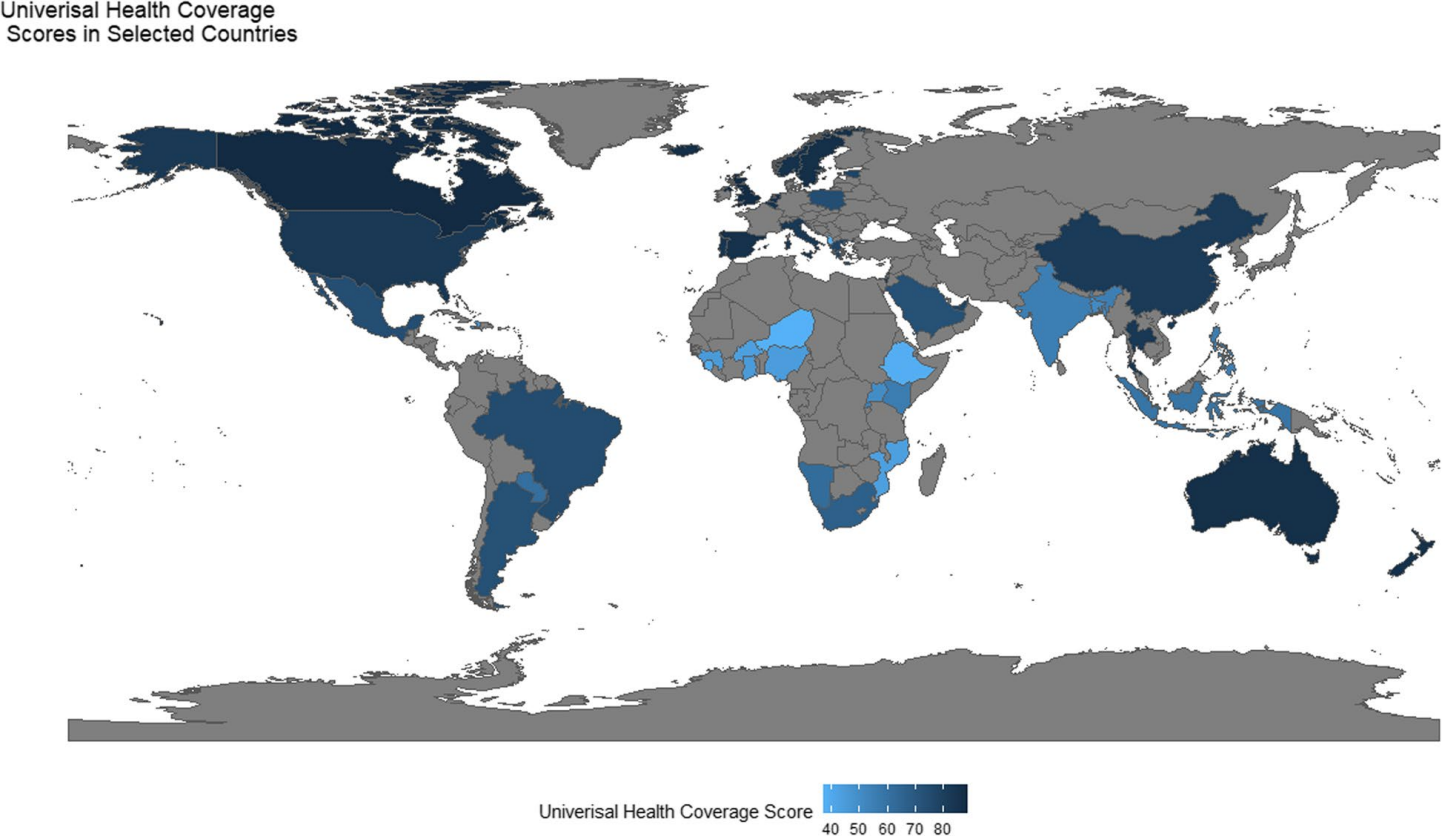
Choropleth map for UHC effective service coverage index in 2019

We can see UHC as an immediate outcome of PHC. The choropleth map shows the UHC effective service coverage index of 45-countries (Fig. 1). The average UHC effective service coverage index was 67.6; the minimum was 37 in Albania and Niger, while the highest value was 89 in Canada. The UHC effective service coverage index value for each country is in the supplementary file (Table S2). Additionally, country-specific progress to specific primary care core principles and long-term health system outcomes.

### Success and weakness

PHC from an accessibility and quality of care point of view scored positive progress per countries contexts. Accessibility matters of how services are available, waiting time to receive care (timeliness), travel time or distance to reach PHC health institutions (geographic accessibility) and the affordability access. Reduced length of hospital stay in the Netherlands [21] and high continuity of care in India [22] was taken as exemplary lessons. Once increased accessibility, a more equitable distribution of health resources was achieved in Kazakhstan [23]. PHC Specialized reference clinics decreased health problem burdens by reducing waiting time and health care cost, and increased client satisfaction in Saudi Arabia [24].

There were an increased number of PHC facilities in Argentina [25]. Australia improved health care services accessibility for prisoners during their release [26]. Primary care was also evaluated for the provision of quality of care. Quality of care was assessed with client satisfaction, services outcome or in a logic-system process. There were diverse achievements of high quality health care for children in Brazil [27] and older people in Poland [28], for immunization, maternal health and epidemic disease control in Saudi Arabia [29], high patient satisfaction in Albania [30] and high patients perceived quality-care in privately owned institutions in Sweden [31].

From cost-effectiveness perspective, an evaluation of the cost-effectiveness of PHC projects in the USA showed that the non-physician service providers ratio were cost-effective [32]. The reason for this difference was not explicitly explained to confirm whether the variation was due to productivity or salary differences. A cost-efficiency measure of PHC in Indonesia showed that community health worker services were less costly than clinic-based care [33] because community services focus on preventive health care. A tool is important to monitor and evaluate the released fund or to generate a new fund. A new health service-related cost monitoring and evaluation tool was developed for fund raising purpose in Bangladesh [9]. High level of coordination, continuity of care and comprehensiveness of PHC in Brazil [34–36], high level of understanding of patient-centredness care in Uganda [37, 38] and presence of better patient-centred care in private clinics in Thailand [39] were successes. India scaled-up comprehensive PHC using ‘Ayushman Bharat’ program in India [10].

There were diverse progress towards narrowing disparity in PHC such as reduced disparities in immigrant populations’ health [40], the presence of inclusive interventions for diverse populations with adequate government budgets in different countries [41] and promotion of health equity (e.g., include equity statement in all health policy) in Australia [42, 43], Canada [44] and in China [45]. Furthermore, policy inclusiveness implemented in some countries through including community engagement in the policy strategy (e.g. Mexico [46], Italy [47] and Kenya [48], engagement of donor agencies and high female representation (e.g., in Nigeria [49, 50] and the UK [51]. Additionally, community oriented and poor-based services in Asia [52] and migrant health volunteer participation in Thailand [53] indicate successful initiation to narrow the gaps. In addition, the higher service readiness has resulted in better effectiveness in Mozambique [54].

There were observed gaps as weaknesses in various countries. For instance, weak continuity care, low accessibility score of comprehensiveness of PHC and community participation in Brazil [34, 55] and a declined continuity of care from 2012 to 2017 in England (due to the unsatisfactory appointment system for patients) [56] wear weakness. Clinics in metropolitan areas and capital cities were less comprehensive as these facilities provided more specialized care and treat medical problems referred from lower health care settings in South Korea [57]. Ineffective PHC reform due to a lack of prior or timely monitoring and evaluation procedures for PHC activities [58] and technical inefficiency in Greek [59], inefficient management in China [60, 61], and lower level of technical efficiency in Spain [62] were weaknesses. PHC services and facility disparities based on geography, education and income status, race, ethnicity and citizenship in Sweden [63, 64], Ghana [65], Nigeria [66], the UK [67, 68] and the UAE [69], South Africa [70], Poland [71] and Brazil [7, 27, 34, 72, 73]. To mention, high population density area in China [74] and people live in far distance did not have access to PHC in Ghana [75]. There was lower service coverage in certified facilities compared to non-certified institutions in Philippines [76].

### Strategies to improve primary health care

There are several leaderships, health workforce, technology, health financing, service delivery and contextual-related strategies and barriers. Transactional and transformational leadership styles [77] facilitated the success of PHC management system. In addition, struggling to shift from a hierarchical to a more relational style in South Africa [78] improved PHC. More comprehensive primary-care improved quality of care and efficiency in the USA [79]. Iceland approached telephone services where no telephone service difference in private and community-owned clinics [80] (Table 1).

**Table 1:** Strategies to improve primary health care

**Effective strategies**	**Countries**
**Leadership**	
Social capital distributive leadership	Canada [81]
Decentralized governance	European countries [82]
Effective technical supervision	Saudi Arabia [29]
Rural community-/family-/school-based healthcare services [83]	Multicounty [83]
Outreach services	Brazil and multicountry [14, 84]
Institutions near to the community	Poland [71, 85], Brazil [86], Niger [87], USA [3], Belgium [88], multicountry [89]
Working with traditional healers	Multicountry [83]
Participatory decision-making processes	South Africa [90]
Contracting with non-governmental partners	Brazil [91] and Bangladesh [92]
Appropriate health care settings	Albania [30]
**Health Financing**	
Financial sustainability	Estonia [93]
Diagonal investment	Ethiopia [94]
**Health workforce**	
Increasing number of well-trained health workers	Estonia [93], multicountry and Brazil [14, 86]
Gender-concordant providers	Multicountry [14]
Train community members/community engagement	Canada [95–97], Spain [98, 99], Australia [100] and South Africa [101]
Skill-mix	South Korea) [57]
Well-functioning Teamwork	Spain [102] and South Africa [103]
**Service delivery**	
After-hours services	UK [104], high-income countries [14], Canada [95, 105]
A strong referral system	Brazil [106]
Scheduling Model’ of care	Brazil [107]
Tools and indicators	Australia and USA [108, 109] and Spain [98, 99]
**Health technology**	
Telemedicine	Brazil [110]
**Client and physician factor**	
Better physician’s and patients’ perception	USA [37, 111, 112]
Patient trust of health care	Uganda [38]

*UK* United Kingdom, *USA* United States of America

### Barriers of primary health care

**Fig. 2:**
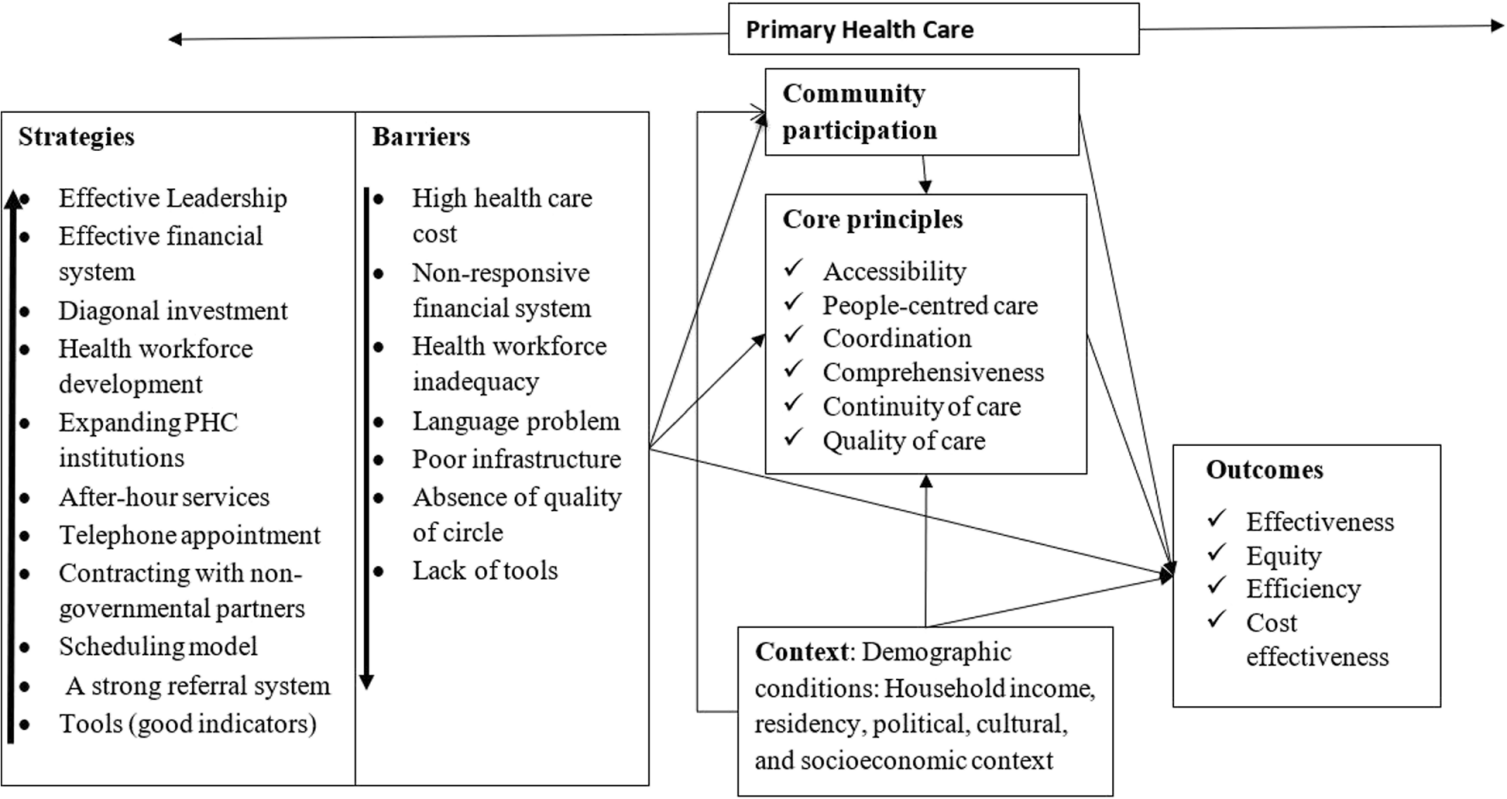
Strategies and barriers of Primary Health Care. Supporting information: additional file, characteristic of studies (Table S1) and UHC effective service coverage index (Table S2)

Principles of PHC affected one another. For example, problem in ‘access’ and ‘non-comprehensiveness services’ [27, 106, 113], uncoordinated care in Brazil [113] and China [114] and continuity of care in China [114] impaired quality of care. Additionally, accessibility problems (unavailability and timeliness [115], financial inaccessibility) in Burkina Faso [116] affects the quality of care. Similarly, a high proportion of walk-in care and high patient volume in Canada [95], problems in accessibility and community orientation in the UK [117] interrupted continuity of care (Table 2). Figure 2 shows the conceptual frameworks to practice, policy, and researchers on the comprehensive PHC based on the main strategies and barriers.

**Table 2:** Barriers of primary health care

**Barriers**	**Countries**
**Leadership**	
Poor infrastructure	Haiti [118] and Australia [119]
Poor organisation	Brazil [115]
Political and legal issue	Brazil [120]
**Health Financing**	
High out-of-pocket payment	Brazil [55, 121, 122], Australia [123], New Zealand [124]
Absence of health insurance	Saudi Arabia [125]
Poor remuneration system	China [114]
Delayed funds	Kenya [48]
**Health workforce**	
Lack of adequate staff	Canada [95, 96, 126–128], Brazil, China, Poland, Australia [72, 114, 129, 130], Belgium [88, 131], Nigeria [49]
Lack of training	Australia [119, 132]
Lack of clear job descriptions	Australia [119], UK [51] and South Africa [133]
Unfair health worker distribution	Ethiopia [134]
**Service delivery**	
Absence of quality of circle	Multicounty [135]
Language carrier	Canada [95, 96, 126–128], Saudi Arabia [29], USA [136, 137]
Lack of tools or guidelines	Multicounty [138], developing countries [9, 139], in Brazil [55, 121, 122], Italy [47], Kenya [48]
Weak client engagement	Australia [119]
Lack of family support	Nigeria [66, 140]
**Client and physician factor**	
Discriminatory perception	Sweden [141]
Poor service hours	Nigeria [66, 140]
PHC services coincidence with market days	Nigeria [66, 140]
Varied perception between clients and providers	South Africa [103]
Attitude of indigenous community	Canada [95, 96, 126–128]
Community’s lack of trust to health service	Australia [129]

*UK* United Kingdom, *USA* United States of America

## Discussion

There was heterogeneous progress towards PHC vision. This review identified effective leadership, financial system, diagonal investment, health workforce development, expanding PHC institutions, after-hour services, telephone appointments, contracting with NGOs, a ‘Scheduling Model and a strong referral system and tools effective strategies to PHC achievement. High health care costs, client’s bad perception to health care, health workers inadequacy, language barrier and lack of quality of circle that barred PHC progress.

The leadership/governance functions greatly impacted PHC. One of its functions is working with NGOs. Working with NGOs improved PHC system because it strengthen the health system [142]. Effective leadership constructing appropriate health care infrastructure expanded municipality areas certainly improves PHC [143] because it would be inclusive to all individuals (e.g., disabled) and up-to-date technologies for health [144, 145]. Effective leadership also allows a bidirectional management system to improve accountability, community participation and support participatory decision-making process in PHC. When people become more responsible, accountability is more likely to be kept in human mind [146]. Effective leaders are also proactive in reviewing health system policy, and monitoring and following health policy inclusiveness [42, 47]. Countries should be curious about their health system reform because ineffective health system reform dismantled the existing PHC system [58, 60]. Health policy reforms depends on how, when and by whom the reform is implemented, and requires public understanding and support, continuous monitoring and evaluation before, during and after implementation[147].

Expanding the municipality or institution of PHC was another effective strategy. The presence of primary health care institutions near to the community can be a prior strategy to PHC performance. It is important in reducing direct, indirect and intangible costs. Walking short distance to health institution reduce transport cost, food cost and productivity loss because clients and care giver (client supporters) can receive service shortly and return to their job. Traveling short distance to health institutions can also prevent/reduce intangible cost, which could happen if clients may not return to work for long time due to long travel. It is supported by providing low-cost services, offering outreach services, providing free transportation to the poor [14, 84] and reaching poor geographical areas improved the accessibility of PHC [89].

A strong Health financing system supported the PHC system. Provision of free transportation to and from PHC institutions to clients (the poor) and availing low-cost services improved PHC [14]. This requires an adequate health budget and sustainable financing [41, 45]. The diagonal investment was a successful strategy for filling the gap due to the comprehensive nature of PHC. A diagonal approach to scale-up of PHC system effectively improved maternal and child health [148]. This approach was also effective in the progress of UHC to care for chronic illness in the overall health system [149].

Adequate health workforce development accelerates PHC progresses [150]. Improving health workforce adequacy, like numbers with different skills, education, engaging interpreters and gender-concordant providers improved PHC. In a country where interpreters were included in the health workforce, PHC performance was improved. However, a PHC system should be careful in recruiting and using interpreters. For example, an interpreter may provide much information to patients with lower English proficiency at a time, but a patient may not grasp all information at once [151]. Gender-concordant health care providers improved PHC. Patient-physician gender concordance might impact patients’ perception (felt treated with respect), especially during sensitive health issues [152]. Despite its effectiveness, the disparity of PHC team composition between regions or institutions, lack of qualified health workers in the community, unbalanced population-to-physician ratio, and health workers’ lack of training interrupted the provision of continuous, coordinated and quality PHC. The absence of a quality circle interrupted PHC continuum of effective progress. In the absence of ‘quality of care circle’, there could be no way to a group of health team who meet regularly to discuss how to adhere with the standard of care, and quality of PHC is disrupted as a result [135]. Inadequate incentives for health workers also impeded accountable health care providers [153].

After-hour service is helpful when medical problems are addressed by few professionals or when health professionals are few due to high health care demands. When working hours are extended beyond eight hours per day, clients can get skilled personnel at PHC centre at any time. As a result, after-hour services reduced demand for acute care and reduced costs [154].

A ‘Scheduling Model’ improved PHC performance through accessibility and quality of PHC by which clients make an appointment to care based on their preference for the type of care and skilled personnel. It also has the power to change the perception of clients whereby clients perceived as they received better care [107]. Similarly, the probabilistic patient scheduling model was effective in a hospital by increasing annual cumulated profit, and decreasing waiting list and waiting times [155]. A scheduling model is an important procedure in a patient-referral system. Approaching this model helps primary care providers not to refer a patients to a physician with numbers of clients on the waiting queue [156].

A strong referral system shape health care system functionality and community perception of care. Moreover, the presence of referral system prevents health care service interruption [157]. In advancing technology, transition from paper-based referral to e-referral system partly solve conundrum of health workforce by using skewed physicians [158].

Telephone access and telephone appointments maintain an effective PHC system. Health technology and supply are the building blocks of health system [159]. Therefore, the absence of health technologies and lack of health system digitalisation lagged behind the successful progress of PHC [61, 121].

The availability of appropriate tools, indicators and data supports the PHC system. Health information-related strategies allow measuring and disseminating health-related data that improves the PHC system [160]. In addition, it is known that offering tools and creating feedback mechanisms for the community reinforce the PHC system [161]. Therefore, a need to have agreed method of PHC cost measurement tool is required, for example, in Australia [6].

Community participation was an effective strategy. It is taken as a specific strategy in capacitating core principles of primary care and improving PHC outcomes. It helps to provide culturally safe care that promotes patients to attend health services for the next care [162]. Community participation improves clients’ perception towards care. In the current review, having better perception and client’s trust to health services supported PHC capacity, whereas bad perception found in contrast.

As to policy implication, a well-functioning health system—health leadership and governance, health finance, appropriate health workforce and availing proper health technology—pushes forward the PHC progress and maintains enacted PHC systems. Researchers can further examine the techniques to solve barriers and advancing emerging strategies. For example, ‘Quality of Circle’, ‘Scheduling Model’ and ‘Diagonal investment’.

## Limitation

Studies exclusively published in English are included in this review. This review might lack the chance of getting more advantageous by including non-English language articles. This scoping review, due to its design nature, lacks a quality appraisal of the included documents, and the current results may need caution in interpretation. Furthermore, a search from a single academic database (PubMed) may miss some important articles in other databases.

## Conclusions

A country with a higher UHC effective service coverage index does not reflect its effectiveness in all aspects of PHC. Strengths of PHC are less costly community health workers services, presence of quality indicators and improved quality of care (e.g., maternal and child health), increased health care coverage, improvement of health outcome due to community participation, provision of comprehensive care and improved resource and service efficiency.

PHC is, beyond the technical practice given at health care spots, a system thinking that entertains multiple strategies towards health system impacts. Continues investment in PHC infrastructure, sustainable financing to reduce health care costs, appropriate workforce planning and training, construction of new PHC institutions in regions of low accessibility and institutionalizing quality of circle will accelerate PHC progress. A valid and agreed measurement tool for PHC attributes is also relevant. Additionally, the research did not address the wholistic concepts of PHC; almost all studies on PHC were only on integrated public and essential health services.

## Supplementary Information

**Additional file 1: Supplementary file 1.** Preferred reporting items for systematic reviews and meta-analyses extension for scoping reviewschecklist.

**Additional file 2: Table S1.** Characteristics of articles.

**Additional file 3: Table S2. **UHC effective service coverage index for countries included in the review.

## Data Availability

The data set is available within this manuscript.

## Abbreviations

NGO: Non-governmental Organisations
PHC: Primary Health Care
UK: United Kingdom
USA: United States of America
