# Supplementary material for: Successes, weaknesses, and recommendations to strengthen primary health care: a scoping review
Source: Arch Public Health. 2023 Jun 2;81:100. doi: 10.1186/s13690-023-01116-0 (PMC10236853; doi:10.1186/s13690-023-01116-0)
Supplement: Supplementary file 2 — Additional file 2: Table S1. Characteristics of articles. [file 13690_2023_1116_MOESM2_ESM.docx]

TableS1: Characteristics of articles

| Author | Publication year | Country (study setting) | Study approach | study population | Dimensions/Attributes | Objective |
| --- | --- | --- | --- | --- | --- | --- |
| Abdullah A et al | 2012 | Indonesia | Qualitative | Stakeholders (from staff to managers) | Cost effectiveness | Develop a costing tool to PHC services (health promotion, sanitation and environment health, maternal and child health and family planning, nutrition, immunization and communicable diseases control, and treatment of common illness) |
| Adie H et al | 2014 | Nigeria | Qualitative | Client and health worker | Community participation | Assess perception and attitude policy and services of PHC |
| Akhavan S. & Tillgren P | 2015 | Sweden | Mixed | client | Equity | Patient experiences and perceptions of equity care in PHC |
| Al-Ahmadi H & Roland M | 2005 | Saudi Arabia | Review | Articles | Quality of care | Examine quality of primary care and factors |
| Aleluia IRS et al | 2017 | Brazil | Qualitative | Document, professionals & managers | Coordination | Assess coordination of care by PHC |
| Alsubaie AM et al | 2001 | Saudi Arabia | Quantitative | client | Accessibility | Observe effect of the main socioeconomic factors affecting patients’ utilization of primary health care services |
| Anderson E et al | 2005 | United Kingdom | Qualitative | health care provider and residents | Community participation | Assess process of public involvement in planning primary health care |
| Arnaudo F et al | 2017 | Argentina | Quantitative | National Population census (all age) | Equity | Degree of equity in the availability of public primary healthcare centers |
| Assefa Y et al | 2018 | Ethiopia | Review | Document | Effectiveness | Assess effectiveness and sustainability of a diagonal investment approach |
| Atting IA et al | 1991 | Nigeria | Quantitative | Community | Accessibility | Assess indicators of Accessibility (proportion of births attended by trained health personnel, proportion of children with diarrhea treated with oral rehydration therapy (ORT), distance from home to regular immunization site, and acceptability of primary health care services to the target population) PHC Coverage |
| Avelino CCV et al | 2015 | Brazil | Quantitative | all age | Quality of care | Quality of the PHC service |
| Battussen R et al | 2002 | Burkina Faso | Qualitative | Clients | Quality of care | User's opinion on the quality of care of PHC services |
| Bath J & Wakerman J | 2015 | Australia | Review | Articles | Community participation | Impact of community participation in primary health care |
| Batisa R et al | 2018 | multicountry | Review | Articles | Equity | Examine two healthcare models, specifically “Primary Medical Care” (PMC) and “Primary Health Care” (PHC) in the context of immigrant populations’ health needs |
| Bousquat A et al | 2017 | Brazil | Mixed | Health worker & client | Coordination | Analyze the healthcare coordination by Primary Health Care (PHC), with the backdrop of building a Health Care Network (RAS) |
| Burström B et al | 2017 | Sweden | Review | Articles | Equity | Impact of current PHC on health inequality |
| Barakat-Haddad C & Siddiqua A | 2015 | UAE | Quantitative | Adolescent | Accessibility | Examined primary health care use and accessibility |
| Carlisle K et al | 2021 | Australia | Qualitative | Observations, health worker, client, community member | Quality of care | Explore the strengths and challenges in QI for Aboriginal and Torres Strait Islander PHC services and their priorities for improvement |
| Catacutan AR | 2005 | Philippines | Quantitative | health facilities | Accessibility | Evaluate service coverage among certified and non-certified facilities |
| Chreim S et al | 2010 | Canada | Qualitative | Stakeholders | Leadership and governance | Understand the dynamics of collective or distributed leadership by attending to change agency roles in a context involving collaboration across health organizations |
| Cleary S et al | 2018 | South Africa | Qualitative | Facilities and leadership team | Leadership and governance | Provide insights into how leadership is currently practiced |
| Comino EJ et al | 2006 | Australia | Quantitative | household survey | Accessibility and Quality of care | explores the utility of population health surveys to address questions relating to access to and use of PHC, using diabetes as an example |
| Cordero-Ferrera JM et al | 2009 | Spain | Quantitative | Population data | Efficiency | efficiency measurement in primary health care with the application of a recently developed method to deal with exogenous variables |
| Costa LB et al | 2021 | Brazil | Quantitative | Client | Quality of care | Quality of PHC |
| Crossland L et al | 2014 | multicountry | Review | Articles | Quality of care | Identify elements that are integral to highquality practice and determine considerations relating to high-quality practice organisation in primary care. |
| Culhane-Pera KA et al | 2021 | USA | Qualitative | Community | Quality of care | amplify the voices of community leaders from seven diverse urban communities view qual‑ity healthcare and fnancial reimbursement based on quality metric scores |
| David J & Zakus L | 1988 | Mexico | Qualitative | Health workers & community | Community participation | Resource dependency and community participation in PHC |
| Davy C et al | 2016 | multicountry | Review | Articles | Accessibility | identify issues that hindered Indigenous peoples from accessing primary health care and then explore how, if at all, these were addressed by Indigenous health care services |
| de Sousa ANA & Shimizu HE | 2021 | Brazil | Quantitative | household survey | Comprehensiveness | Analyze comprehensiveness elements in Primary Care |
| Desta BF et al | 2020 | Ethiopia | Quantitative | Districts | Leadership and governance | compare district level capacity and performances between leadership, management and governance (LMG) and non-LMG districts. |
| Dewulf B et al | 2013 | Belgium | Quantitative | household survey | Accessibility | verify whether these population-to-physician ratio methods are accurate enough for adequately designating medical shortage areas and explore how these perform relative to more advanced GIS-based methods. |
| Dietrich AJ & Olson AL | 1981 | India & Mexico | Quantitative | Client and health worker | Continuity | Evaluate continuity with a personal provider |
| Espinosa-Gonzalez AB et al | 2019 | European | Quantitative | stakeholders | Leadership and governance | Analyze the interactions between PHC functions and their impact in PHC delivery, particularly in providers’ behaviour and practice organization |
| Cordero-Ferrera JM et al | 2013 | Spain | Quantitative | Population data | Efficiency | Extend the literature on measuring efficiency in primary health care by considering the influence of quality indicators and environmental variables conjointly in a case study |
| Field KS et al | 2002 | United Kingdom | Quantitative | Clients & health worker | Accessibility | Socio-economic and locational determinants of accessibility and utilization of primary healthcare |
| Figueiredo DCMM et al | 2018 | Brazil | Quantitative | Clients | Quality of care | Describe the evaluation of patients that participated in the National Program for Improving the Access and Quality in Primary Health Care for the comprehensive healthcare, the bond and the coordination of care in the country’s macro-regions |
| Gage Adet al | 2018 | Haiti | Quantitative | household survey | Quality of care | How facility infrastructure and the quality of primary health care service delivery were associated with community utilization of primary health care services |
| Gonçalves MR et al | 2016 | Brazil | Quantitative | Patients | Quality of care | Investigate the relation of hospitalization for ambulatory care sensitive conditions (ACSC) with the quality of public primary care health services |
| Goni S | 1999 | Spain | Quantitative | Teams (health worker) | Effectiveness | Observe how the introduction of one tool traditionally used in the private sector, the organisation of work through teams, can contribute to improved performance in public health services. |
| Hadded S et al | 1998 | Guinea | Qualitative | Community | Quality of care | Identify, characterize, and classify the criteria that the public uses to judge the quality of primary health care (PHC) services |
| Haggerty JL et al | 2008 | Canada | Quantitative | Clients and health worker | Accessibility, continuity, coordination | Identify attributes of clinic organization and physician practice that predict accessibility, continuity, and coordination of care as experienced by patients |
| Hove J et al | 2021 | South Africa | Review | Articles | Community participation | Provide evidence on forms, extents, contexts and dynamics of community participation in primary health care (PHC) and water governance |
| Heard A et al | 2013 | Bangladesh | Quantitative | Health facilities, households | Accessibility, cost effectiveness, quality of care | Evaluate a large, ongoing effort to improve urban primary health care (PHC) in Bangladesh through expansion of publicly funded urban health facilities and contracting with partner non-governmental organisations (NGOs) |
| Higgs G et al | 2018 | United Kingdom | Quantitative | household survey | Accessibility | Examine variations in geographical accessibility to general practitioner (GP) surgeries offering appointment times outside of ‘core’ operating hours. |
| Isaksson D et al | 2016 | Sweden | Quantitative | Geographical site, where HF located | Equity | Examine how the primary care choice reform has affected geographical equity by analysing patterns of establishment on the part of new private providers. |
| Iyanda OF & Akinyemi | 2017 | Nigeria | Qualitative | Community and health worker | Community participation | Assess the process indicators and other factors influencing community participation in the delivery of primary health care |
| Jatrana S & Crampton P | 2009 | New Zealand | Quantitative | household survey | Accessibility | Examined the demographic, socioeconomic, health behaviour and health determinants of financial barriers to access to general practitioner services, prescription drugs and dental care in New Zealand (NZ). |
| Joshi C et al | 2013 | Multicounty | Review | Articles | Accessibility, quality of care, coordination | Identify the components of primary health care service delivery models for such populations which have been effective in improving access, quality and coordination of care |
| Kaag MEC et al | 1996 | Netherlands | Quantitative | Patients | Quality of care | Assessing quality of care of shorted hospital stays with home care by the community nurse and/or the genral practitioner (GP) |
| Khakh AK et al | 2018 | Canada | Quantitative | Geographical site and HF | Accessibility | Assess spatial accessibility of PHC |
| Kila´nska D et al | 2021 | Poland | Quantitative | Medical record | Accessibility | Assess the impact of structural changes which increased the nurses’ competences on the accessibility to prescription visits for patients receiving primary healthcare on the example of Medical and Diagnostic Centre, and to discuss the general trend of legal changes in nursing profession regulations |
| Kironde S & Kahirimbanyi M | 2002 | South Africa | Quantitative | community & health worker | Community participation | Explore the feasibility of community participation in a high-burden Tuberculosis Control Programme and to establish how supervision of treatment by lay volunteers compares with other methods of tuberculosis treatment delivery |
| Krztoń-Królewiecka A et al | 2016 | Poland | Quantitative | Health worker | Quality of care, accessibility, comprehensiveness, continuity, coordination, efficiency, equity, cost effectiveness | Analyze the insight of nine core dimensions of Polish PC system: “Economic conditions”, “Workforce”, “Accessibility”, “Comprehensiveness”, “Continuity”, “Coordination”, “Quality of care”, “Efficiency” and“Equity” and to identify the characteristics of the providing physicians that influence their perception of the quality of care |
| Kurpas D et al | 2013 | Poland | Quantitative | Patients | Quality of care | Evaluate correlations between patients’ characteristics and their assessments of the quality of health care and to identify the primary factors influencing the quality of care for chronically ill patients. |
| Langford M et al | 2016 | United Kingdom | Quantitative | household survey | Accessibility | Multi-modal two-step floating catchment area analysis of primary health care accessibility |
| Lapão LV et al | 2017 | Portugal | Qualitative | Documents and articles | Coordination | Compare the organization of the Primary Healthcare from both regions, searching to identify the advancement which in terms of the Delivery Health Networks’ coordination |
| Lechowski L & Jasion A | 2021 | Poland | Qualitative | Geographic site & HF | Accessibility | Assess the spatial accessibility of basic and universal healthcare (understood as primary healthcare (PHC) facilities |
| Leng Y et al | 2019 | China | Quantitative | Documents and records | Efficiency | Assess the impact of the China’s health care reform policy in 2009 on the intangible service efficiency of PHC institution (PHCI) and exploring the way to improve the service efficiency of PHCI. |
| Li Xi et al | 2020 | China | Review | Articles, expert, practitioner | Quality of care | Identify the causes for this poor quality, and provide policy recommendations |
| Llyoyd JE et al | 2017 | Australia | Qualitative | Community | Accessibility | Assess how primary health care can better meet the health care and social support needs of Aboriginal Australians transitioning from prison to the community |
| Luis ADA | 2016 | Mozambique | Quantitative | geography, population, HF | Accessibility | Measure the geographic accessibility of population to existing Healthcare Centers (HC), and to estimate the number of persons served by the health network |
| Luisi D & Hämel K | 2020 | Italy | Qualitative | Document | Community participation | Explores the evolution of the meaning and conceptualization of community participation and empowerment in police |
| Lum ID et al | 2016 | Canada | Qualitative | community (immigrant) | Accessibility | Examine the lived experiences of immigrants living in a small urban centre with regards to the primary healthcare system |
| Luna I & da Silva MT | 2015 | Brazil | Qualitative | health worker | Coordination | Describe and explain the work coordination process applied by multi-professional teams to deal with the intangibility inherent in the healthcare services |
| Mandel MD et al | 2003 | Israeli | Quantitative | health worker & HF | Quality of care | Analyze the quality of primary care |
| Marques JF et al | 2018 | Brazil | Quantitative | health facility | Accessibility | Assess the physical accessibility from the front desk of primary healthcare units |
| Maun A et al | 2015 | Sweden | Quantitative | Clients | Quality of care | Is the quality of primary healthcare services influenced by the healthcare centre’s type of ownership? |
| Meads G et al | 2017 | Latin America and Northwest Europe | Review | Articles | Leadership and governance | Assess community governance in PHC |
| Mercer A et al | 2004 | Bangladesh | Quantitative | household/population | Effectiveness | Effectiveness of a non-governmental organization (NGO) primary health care programme |
| Mogakwe LJ et al | 2019 | South Africa | Qualitative | Stokeholds | Quality of care | Describe recommendations to facilitate managers’ compliance with quality standards of PHC clinics |
| Mudrick et al | 2012 | USA | Quantitative | Health facilities | Accessibility | Describe overall primary care office physical accessibility and identify (1) in which areas offices meet access criteria, (2) which accessibility criteria are most often not met, and (3) whether there are urban/non-urban differences |
| Mukiapini S et al | 2017 | South Africa | Mixed | health workers & clients | Effectiveness | Assess PHC team effectiveness and PHC organisation and performance. |
| Murillo-Zamorano LR & Petraglia C | 2011 | Spain | Quantitative | Medical record | Efficiency | Assess technical efficiency in primary health care |
| Novignon J & Nonvignon J | 2017 | Ghana | Quantitative | Health facilities | Efficiency | Estimate efficiency among primary health facilities (health centers), examine the potential fiscal space from improved efficiency and investigate the efficiency disparities in public and private facilities |
| Oikonomou N et al | 2015 | Greece | Quantitative | health facilities | Efficiency | Measure the efficiency of the rural Health Centres |
| Okoronkwo IL et al | 2014 | Nigeria | Quantitative | Clients | Accessibility | Assess patterns of utilization of PHC services among adults |
| Oliphant NP et al | 2021 | Niger | Quantitative | Health facilities & health workers | Accessibility | Assess geographical accessibility of primary healthcare services at community level and strategies for optimising geographical accessibility to these services |
| Oosterveer TM & YoungTK | 2015 | Canada | Qualitative | Health care provider, clients & community | Accessibility | Analyze the challenges in accessing PHC services by indigenous people in remote communities |
| Paré-Plante A et al | 2018 | Canada | Quantitative | Health care provider & clients | Accessibility | Assess which PHC organizations’ characteristics are associated with improved first-contact accessibility |
| Philips H et al | 2014 | Belgium | Quantitative | Community | Accessibility | Examine how primary health care can be made accessible to vulnerable groups |
| Pinto A et al | 2021 | Brazil | Quantitative | Health facility | Accessibility | Assessment of the accessibility of primary health care facilities |
| Pinto LF et al | 2016 | Brazil | Quantitative | clients | Quality of care | Evaluate the extent of PHC attributes |
| Pocas KC et al | 2017 | Brazil | Quantitative | Health facilities | Accessibility | Potential coverage indicators were estimated for infrastructure, health team composition and services available |
| Rabelo ALR et al | 2019 | Brazil | Quantitative | Health worker & client | Continuity and coordination | Evaluate the attributes of primary health care, care coordination and longitudinality, from the perception of the professional and patients |
| Richard L et al | 2016 | multicounty | Quantitative | Stakeholders (Expert, researchers, leaders) | Equity | Equity of access to primary healthcare for vulnerable populations |
| Rifkin SB | 1987 | Asia | Review | Urban poor | Community participation | Assess why participate? who participates? how do they participate? |
| Rocha ESC et al | 2020 | Brazil | Quantitative | Health worker | Accessibility, continuity, coordination, comprehensiveness, community participation | Assess the attributes of Primary Health Care from the perspective of health professionals, comparing services in the Special Indigenous Health District and the Municipal Health Offices |
| Rohrbasser A et al | 2018 | multicounty | Review | articles | Quality of care | Identify possible definitions of quality circles, their origins, and reported effectiveness in primary health care, and to identify gaps in our knowledge |
| Rossignol M et al | 2000 | Canada | Quantitative | Health workers and clients | Coordination | Measure the effectiveness of the coordination of PHC  program as a mean for implementing clinical practice guidelines for low-back pain in an urban community |
| Salisbury C et al | 2009 | United Kingdom | Quantitative | health workers | Continuity | Assess whether organisational changes have had an impact on continuity, it is necessary to define and measure the term |
| Saric J et al | 2021 | Albania | Quantitative | Health facilities, health worker, patient | Quality of care | Compare quality of care before and after the 4 years of health for all project implementation in PHC |
| Scarborough J et al | 2015 | Australia | Review | health facilities | Equity | Suggest ways of increasing the cohesiveness of national primary healthcare strategies and hepatitis C strategies, with the aim of ensuring that all these strategies include ways to address barriers and facilitators to access to primary healthcare and equity for people with hepatitis C |
| Schmidt B et al | 2016 | Australia | Qualitative | health worker, clients, stakeholder | Coordination | Explore how a client-centred Chronic Care model was implemented by Indigenous Health Workers (IHWs) at participating sites in a trial of IHW-led case management and understand the experiences of engaging with the model from the perspective of the IHWs, health team members and clients. |
| Sigurdsson JA et al | 1988 | Iceland | Quantitative | clients | Accessibility | Accessibility by telephone of primary health care |
| Singh D et al | 2021 | India | Quantitative | health facility | Cost effectiveness | Assess the cost of this scale-up to inform decisions on budgetary allocation, as well as to set the norms for capitation-based payments |
| Sirilak S et al | 2012 | Thailand | Mixed | health worker, clients, stakeholder | Community participation | Explores the identities of volunteers, their relationship with program management, and their attitudes. The study also investigates the impact of the volunteers, from the migrants’ and healthcare workers’ perspective |
| Sitienei J et al | 2021 | Kenya | Qualitative | health worker, client | Community participation | Examined the implementation of community participation, through collaborative governance in primary health care facilities |
| Sola GJI et al | 2016 | Spain | Quantitative | PHC managers | Leadership and governance | Describe and learn about the self-perception of behaviours and leadership styles among PHC managers; to determine the influence of the leadership style on job satisfaction, efficiency, and willingness to work in a team; and to determine the relationship between transformational and transactional styles according to age, gender, profession, type of manager years of management experience, and the type of organization |
| Spike EA et al | 2011 | Australia | Qualitative | health worker, community, stakeholder | Accessibility | Determine whether community-based asylum seekers experience difficulty in gaining access to primary healthcare services, and to determine the impact of any difficulties described. |
| Tanser F et al | 2006 | South Africa | Quantitative | clients | Accessibility | Investigate differences in rural, urban and periurban usage patterns of clinics in the study area and to quantify the effect of physical access to clinic on usage. |
| Thomas SL et al | 2015 | Australia | Qualitative | Stakeholders | Equity | Define the population thresholds governing which PHC services would be best provided by a resident health worker, and to outline attendant implementation issues. |
| Uchôa SADC et al | 2016 | Brazil | Quantitative | health worker | Accessibility | Analyze the influence of contextual indicators on the performance of municipalities regarding potential access to primary health care |
| Vidal TB et al | 2019 | Brazil | Quantitative | clients | Quality | Evaluate whether the scheduling model influences the perception of the user about the quality of primary health care centers |
| Vieira-da-Silva LM et al | 2010 | Brazil | Quantitative | Clients and health worker | Accessibility | Evaluate the results of a project designed to improve accessibility to healthcare services |
| Wiese M et al | 2011 | Australia | Qualitative | Health worker | Coordination | Investigate the current links between GPs and local primary healthcare providers |
| Woldemichael A et al | 2019 | Ethiopia | Quantitative | household survey | Equity | Assess availability and measure magnitude and trend of inequalities in accessibility of health centre based PHC resources |
| Wong S & Regan S | 2009 | Canada | Quantitative | community | Accessibility, continuity and efficiency | Examining how to deliver primary health care (PHC) services and increase their accessibility (regardless of geographic location) from the patient’s perspective is needed |
| Wong ST et al | 2014 | Canada | Mixed | Literature, clients, health worker | Equity | Develop a core set of indicators that could be used for measuring and monitoring the performance of primary health care organizations’ capacity and strategies for enhancing equity-oriented care |
| Wright M et al | 2021 | Australia | Quantitative | Reports | Cost effectiveness | Explore how much of Australia’s national health expenditure is allocated specially to general practice services, and more broadly to primary healthcare (PHC) services. |
| Zhang X et al | 2013 | China | Quantitative | Documents/reports | Equity | Analysis of government investment in primary healthcare institutions to promote equity during the three-year health reform program |
| Zhang Y et al | 2018 | China | Quantitative | Reports | Equity and efficiency | Evaluate the changes of equity and efficiency in PHC resource allocation (PHCRA) and explored ways to improve the current situation |
| Maele NV et al | 2019 | Low-and Middle-income countries | Quantitative | Reports (The system of health accounts) | Cost effectiveness | Tested and examined different measurement options using the System of Health Accounts (SHA) 2011 for systematic monitoring of PHC expenditure |
| Fleming NS et al | 2006 | USA | Quantitative | Clients and health workers | Patient-centredness | Evaluate the quality of care and financial impact of the ambulatory electronic health record implementation |
| Henry SG et al | 2016 | USA | Quantitative | Clients and health worker | Patient-centredness | Investigate associations between ratings of “thin slices” from recorded clinic visits and perceived patient-centeredness |
| Mann C et al | 2018 | England | Qualitative | Health worker | Patient-centredness | Evaluate the effect on patient-centredness of a novel computer template used in multimorbidity reviews |
| Mayer SD et al | 2016 | USA | Quantitative | Clients | Patient-centredness | Examined the perceptions of care team empathy and patient-centeredness between English- and Spanish-speaking patients |
| Pongsupap Y and Lerberghe WV | 2006 | Thialand | Quantitative | Clients | Patient-centredness | Document differences in provider behaviour between private and public providers in hospital outpatient departments, health centres and clinics |
| Rose GL et al | 2021 | USA | Quantitative | Clients | Patient-centredness | Describe the development of the Patient Centeredness Index (PCI), evaluate its psychometric characteristics and evaluate the relationships between scores on the PCI and an established measure of empathy |
| Roter DL et al | 2020 | USA | Quantitative | Clients and health worker | Patient-centredness | Explore consequences of interpreter mediation of visit communication on patient  centered dialogue and patient satisfaction with interpreter listening |
| Waweru E et al | 2020 | Uganda | Mixed | Clients | Patient-centredness | Highlight patient perspectives by show casing their perceptions of their experience of patient-centred care at primary health facilities |
| Kim HJ et al | 2021 | South Korea | Quantitative | Clinics | Comprehensiveness | assessed comprehensiveness and factors for each clinic as the number of SMDGs treated in each clinic for 2 years |
| O'Malley AS et al | 2019 | USA | Quantitative | Health workers | Comprehensiveness | Develop claims‐based measures of comprehensiveness of primary care physicians (PCPs) and summarize their associations with health care utilization and cost |
| Myloneros T and Sakellariou D | 2021 | Greece | Review | Articles | Effectiveness | Examine whether the Primary Health Care reforms during that period assisted the country in moving towards Universal Health Coverage |
| Tammes P et al | 2021 | England | Quantitative | General practitioner-patient survey data | Continuity | Confirm reports of declining continuity of care, explore differences in decline according to practice characteristics, and examine associations between practice populations or appointment provision and changes in continuity of care. |
| Meiesaar K & Lember M | 2004 | Estonia | Quantitative | Document | Efficiency | Assess the efficiency and sustainability of using health resources in Estonian primary health care |
| Begley CE et al | 1989 | USA | Quantitative | Record/document | Cost effectiveness | Assess a cost-effectiveness evaluation of primary health care projects for the poor |
| Berman P | 1989 | Indonesia | Quantitative | Record/document | Cost effectiveness | Are certain types of small, rural health facilities more efficient than others in providing outpatient contacts? |
| Acquah-Hagan G et al | 2022 | Ghana | Quantitative | Vulnerable populations | Accessibility | Assess availability and Affordability of Primary Health Care Among Vulnerable Populations in Urban Kumasi Metropolis: Family Health Perspective |
| Shaltynov A et al | 2022 | Kazakhstan | Quantitative | Records/secondary data | Accessibility and inequality | Assessment of primary healthcare accessibility and inequality in north-eastern Kazakhstan |
| Alshowair A et al | 2022 | Saudi Arabia | Quantitative | Patients, health care provider | Effectiveness and cost effectiveness | Assessment of Primary Health Care Specialized Reference Clinics in Riyadh First Health Cluster: |
| Chauhan AS et al | 2022 | India | Quantitative | PHC institutions | Cost effectiveness | Estimate the cost of providing healthcare services at sub centre (SC) and primary health centre (PHC) level in four selected States of India |
| Jia P et al | 2022 | China | Quantitative | PHC institutions | Accessibility | Inequalities of spatial primary healthcare accessibility in China |
| Pope S et al | 2022 | Mozambique | Quantitative | PHC institutions | Effectiveness | Primary Health Care Management Effectiveness as a Driver of Family Planning Service Readiness |
| Gizaw Z et al | 2022 | Not specific settings | Systematic review | Secondary data (articles) | Accessibility | To identify key approaches from international experiences to enhance access to PHC services in rural communities |
