# Supplementary material for: Successes, weaknesses, and recommendations to strengthen primary health care: a scoping review
Source: Arch Public Health. 2023 Jun 2;81:100. doi: 10.1186/s13690-023-01116-0 (PMC10236853; doi:10.1186/s13690-023-01116-0)
Supplement: Supplementary file 3 — Additional file 3: Table S2. UHC effective service coverage index for countries included in the review. [file 13690_2023_1116_MOESM3_ESM.docx]

Table S2: UHC effective service coverage index for countries included in the review

| Countries | UHC effective service coverage 2019 |
| --- | --- |
| Albania | 37 |
| Argentina | 73 |
| Australia | 87 |
| Bangladesh | 51 |
| Belgium | 85 |
| Brazil | 75 |
| Burkina Faso | 43 |
| Canada | 89 |
| China | 82 |
| Estonia | 78 |
| Ethiopia | 38 |
| Ghana | 45 |
| Greece | 78 |
| Guinea | 45 |
| Haiti | 47 |
| Iceland | 87 |
| India | 55 |
| Indonesia | 59 |
| Israeli | 84 |
| Italy | 83 |
| Kenya | 56 |
| Mexico | 74 |
| Mozambique | 43 |
| Namibia | 62 |
| Netherlands | 86 |
| New Zealand | 86 |
| Niger | 37 |
| Nigeria | 44 |
| Norway | 86 |
| Paraguay | 61 |
| Philippines | 55 |
| Poland | 74 |
| Portugal | 84 |
| Rwanda | 54 |
| Saudi Arabia | 73 |
| Sierra Leone | 39 |
| South Africa | 67 |
| South Korea | 87 |
| Spain | 86 |
| Sweden | 87 |
| Thailand | 83 |
| UAE | 78 |
| Uganda | 50 |
| United Kingdom | 88 |
| USA | 83 |
